# The Prevention of Unnecessary Blindness

**Published:** 1907-03

**Authors:** 


**A Monthly Review of Medicine and Surgery.**

*EDITOR:*

WILLIAM WARREN POTTER, M. D.

All communications, whether of a literary or business nature, books for review and exchanges, should be addressed to the editor 238 DELAWARE AVE., BUFFALO, N. Y.

VOL. LXII. MARCH, 1907. No. 8

### The Prevention of Unnecessary Blindness

IF it be true, and there seems to be little doubt of it, that less than thirty per cent. of all cases of blindness are absolutely unavoidable and that by the adoption of practical measures this calamity might be averted in a large number of instances, the subject is one of more than medical interest; it becomes at once one of great public importance. It has long been known that gonococci conjunctivitis contributes enormously to this needless loss of sight. Just what proportion of eyes are lost from this cause it is difficult to determine. The most careful figures seem to be those of Magnus who in 2528 cases of blindness found 10.86% due to ophthalmia neonatorum.

The British Royal Commission on the Condition of the Blind estimated that there were about 7000 persons in the United Kingdom who had lost their sight from this disease, or about 22 per cent. of the entire blind; while Ramos says that in Mexico the common cause of blindness is ophthalmia neonatorum, and that in round numbers 4500 or about 30 per cent. owe their blindness to this disease. It is gratifying, therefore, that the American Medical Association has taken up this subject seriously, as will be seen from a circular letter published elsewhere in this issue, in endorsement of Cohn's pronouncement "that this disease must and shall disappear before the advance of civilisation." Significant, too, is the fact that for the first time the state recognises the economic importance of preventing unnecessary blindness and thereby limiting pauperism. The open letter of the commission covers a wider range of subjects. The deadly toy pistol and other Independence Day explosives and methyl alcohol, which is a cause of optic nerve atrophy, have already engaged the attention of the medical profession. That the inquiries which are being instituted should give data of great and permanent value as a basis for protective legislation, cannot be doubted. Of almost equal importance are the investigations on which the commission is employed concerning the ability of the blind to engage in productive work. As Helen Keller has said:

> "There is no law on the statute books compelling people to move up closer on the bench of life to make room for a blind brother; but there is a divine law written on the hearts of men constraining them to make a place for him not only because he is unfortunate, but also because it is his right as a human being to share God's greatest gift, the privilege of man to go forth unto work."

Unfortunately, the appropriation for doing the important work which the commission was directed to perform was inadequate for its purpose and a supplemental bill has been introduced into the legislature asking for a further appropriation of five thousand dollars. There should be no hesitation whatever on the part of the legislature in promptly passing this bill. Similar permanent commissions for the blind have been created in Massachusetts and in Maine, in the latter state with an appropriation of twenty thousand dollars for carrying on its work. The unpaid labors of the men engaged in these important investigations should receive the seal of prompt approval, for the valuable results accruing from their efforts cannot be estimated either from a humanitarian or an economic viewpoint.

